# Supplementary material for: Cu-Doped KCl Unfolded Band Structure and Optical Properties Studied by DFT Calculations
Source: Materials (Basel). 2020 Sep 26;13(19):4300. doi: 10.3390/ma13194300 (PMC7579127; doi:10.3390/ma13194300)
Supplement: Supplementary file 1 [file materials-13-04300-s001.pdf]

Cu-Doped KCl Unfolded Band Structure and Optical Properties Studied by DFT Calculations.

César Castillo-Quevedo [1], Jose Luis Cabellos [2,*], Raul Aceves [2,], Roberto Núñez-González [3] and Alvaro Posada-Amarillas [2,*]

[1] Departamento de Fundamentos del Conocimiento, Centro Universitario del Norte, Universidad de Guadalajara, Carretera Federal No. 23, Km. 191, C.P. 46200, Colotlán, Jalisco, México; castillo.quevedo@cunorte.udg.mx

[2] Departamento de Investigación en Física, Universidad de Sonora, Blvd. Luis Encinas y Rosales S/N, 83000 Hermosillo, Sonora, México; raul.aceves@unison.mx

[3] Departamento de Matemáticas, Universidad de Sonora, Blvd. Luis Encinas y Rosales S/N, 83000 Hermosillo, Sonora, México; ronunez@mat.uson.mx

* Correspondence: jose.cabellos@unison.mx (J.L.C.), posada@cifus.uson.mx (A.P.-A.)

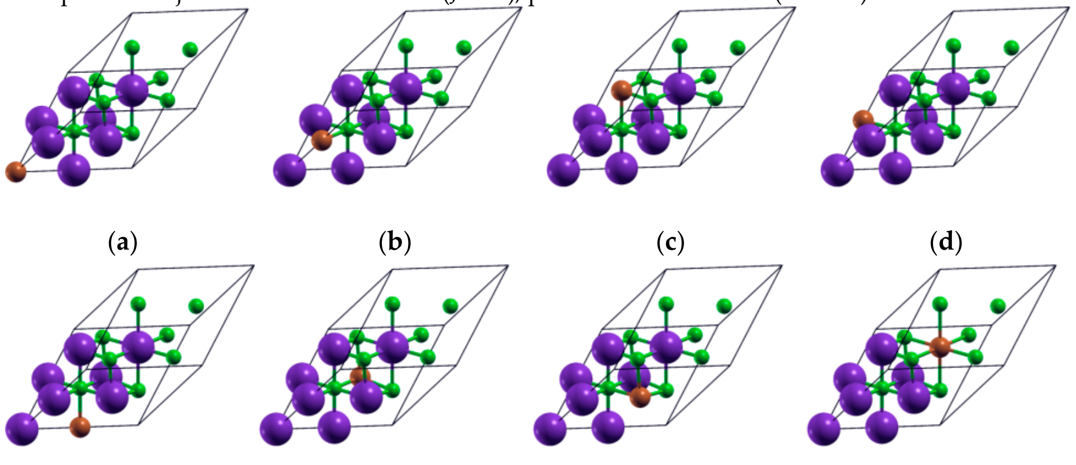

(**a**) (**b**) (**c**) (**d**)

(**e**) (**f**) (**g**) (**h**)

Figure S1. From (**a**) to (**h**) supercell of KCl doped with Cu atom with 16 atoms at the base. we considerer the dopants occupy only the substitutional sites replacing all host K cations. The violet-, green-, and copper-colored spheres represent the potassium, chlorine, and copper atoms, respectively.

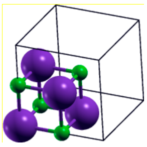

Figure S2. Cubic supercell of pure KCl with eight atoms at the base. The relation between cells FCC-PC, shows in Figure 1a, and cubic SC with eight atoms basis, is a geometric factor of 1.4142. The computed lattice constant in the FCC-PC 4.507 Å, we can calculate the lattice constant for cubic-SC employing the factor 1.4142. The violet- and green-colored spheres represent the potassium and chlorine atoms, respectively.

Table S1. List of non-equivalent atoms in the unit cell (cryst. Coords.) of the KCl:Cu system.

# nat	x	y	z	Name	Z
1	0.0000000000	0.0000000000	0.0000000000	Cu	29
2	0.5000000000	0.0000000000	0.0000000000	K	19
3	0.5000000000	0.5000000000	0.5000000000	K	19
4	0.2500000000	0.2500000000	0.2500000000	Cl	17

5	0.7765725826	0.2234274174	0.2234274174	Cl	17

Table S2. List of atoms in the unit cell (cryst. coords.) of the KCl:Cu system. Molecular formula: Cu(1) K(6) K(1) Cl(2) Cl(6). Number of non-equivalent atoms in the unit cell: 5. Number of atoms in the unit cell: 16. Number of electrons: 298.

# nat	x	y	z	Name	Z
1	0.0000000000	0.0000000000	0.0000000000	Cu	29
2	0.5000000000	0.0000000000	0.0000000000	K	19
3	0.0000000000	0.0000000000	0.5000000000	K	19
4	0.0000000000	0.5000000000	0.5000000000	K	19
5	0.5000000000	0.5000000000	0.0000000000	K	19
6	0.5000000000	0.0000000000	0.5000000000	K	19
7	0.0000000000	0.5000000000	0.0000000000	K	19
8	0.5000000000	0.5000000000	0.5000000000	K	19
9	0.2500000000	0.2500000000	0.2500000000	Cl	17
10	0.7500000000	0.7500000000	0.7500000000	Cl	17
11	0.7765725826	0.2234274174	0.2234274174	Cl	17
12	0.2234274174	0.7765725826	0.7765725826	Cl	17
13	0.7765725826	0.7765725826	0.2234274174	Cl	17
14	0.2234274174	0.2234274174	0.7765725826	Cl	17
15	0.2234274174	0.7765725826	0.2234274174	Cl	17
16	0.7765725826	0.2234274174	0.7765725826	Cl	17

Table S3. Lattice vectors (bohr). The lattice parameters (bohr): 15.855582 15.855582 15.855582, The lattice parameters (ang): 8.390413 8.390413 8.390413, and The lattice angles (degrees): 60.000 60.000 60.000.

	x	y	z
1	15.8555818239	0.0000000000	0.0000000000
2	7.9277909119	13.7313366513	0.0000000000
3	7.9277909119	4.5771122171	12.9460283478

In Table S1, Table S2, and Table S3 is given the list of non-equivalent atoms in the unit cell, the list of atoms in the unit cell and the three lattice vectors, respectively, of the KCl: Cu system.

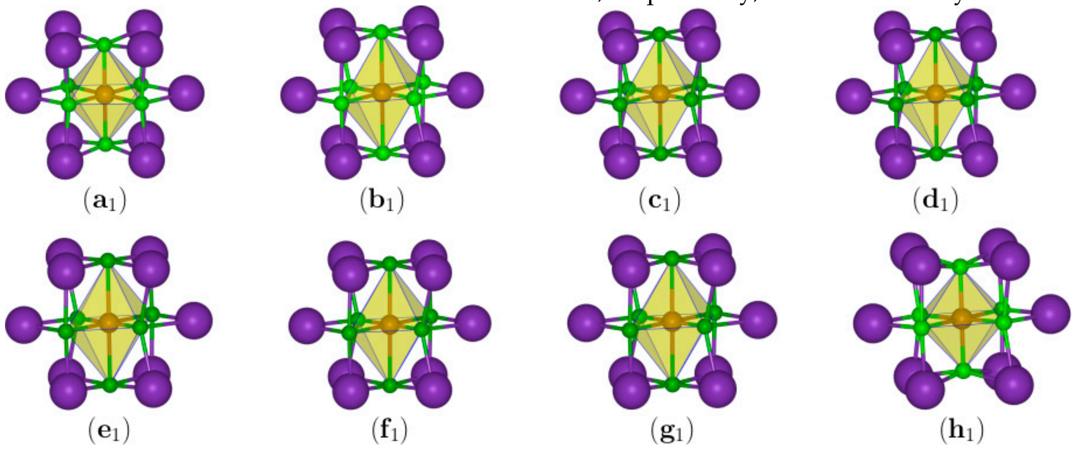

Figure S3. The panel (**a**1) and panel (**h**1) show the octahedral square bipyramidal shape. In this geometry, the six Cl atoms are symmetrically arranged around a central Cu atom. The optimized bond length between the Cu atom and the six surrounding Cl atoms is 2.651 Å. The panel (**b**1–**g**1) shows slightly twisted octahedra square bipyramidal shape. In this octahedrons, there are four optimized bound lengths of Cu-Cl of 3.146 Å and two of 2.151 Å. The elongation of four bounds, in the slightly twisted octahedron square bipyramidal shape, could be related to a Jahn–Teller effect, and the distorted system could be more stable than the undistorted one. The violet-, green-, and copper-colored spheres represent the potassium, chlorine, and copper atoms, respectively.

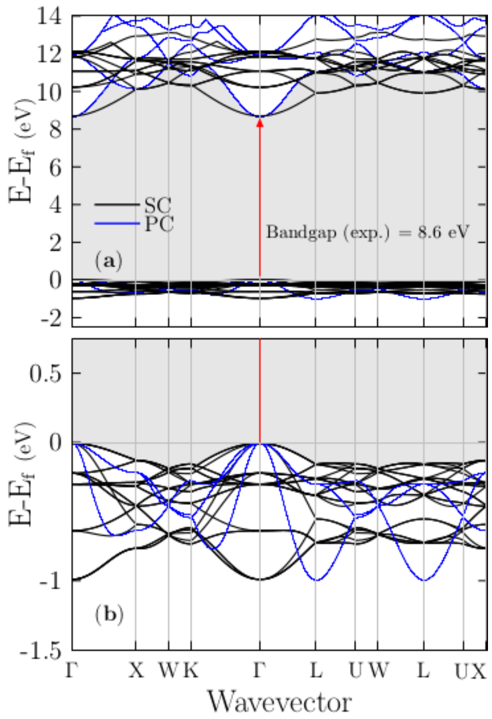

Figure S4. The upper panel (**a**) shows the band structure of the primitive cell (PC) for KCl. (blue lines) and the folded band structure of the SC of pristine K_8Cl_8 (black lines) along with the same high-symmetry directions of the Brillouin zone (BZ) of the PC. The lower panel (**b**) shows a zoomed view in the region −1.5 to 0.75 eV. The scissors corrected direct bandgap indicated by the red arrow is 8.6 eV at the Γ point. The effect of the scissors operator is a rigid shift of the bandstructure to experimental value. The blue lines correspond to the PC band structure, and the black lines correspond to the SC band structure.

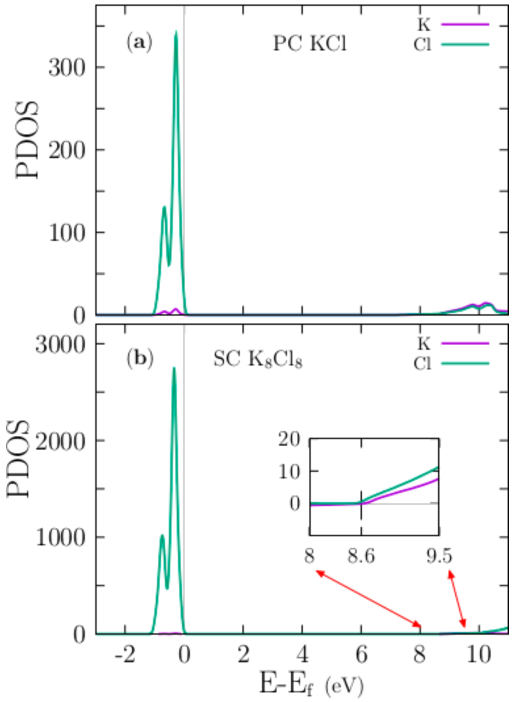

Figure S5. The upper panel (**a**) shows the scissors corrected projected density of states (PDOS) for the pristine PC for KCl. The lower panel (**b**) shows the scissors scissors corrected PDOS for the pristine SC of K_8Cl_8. In both plots, the Cl-3p states dominate at the Fermi level. The inset in the lower panel (**b**) shows a zoomed view in the range 8 to 9.5 eV energy axes, clearly shows that density of states start to raise at experimental energy range of 8.6 eV, as we expected. The color code is violet for K and green for Cl.

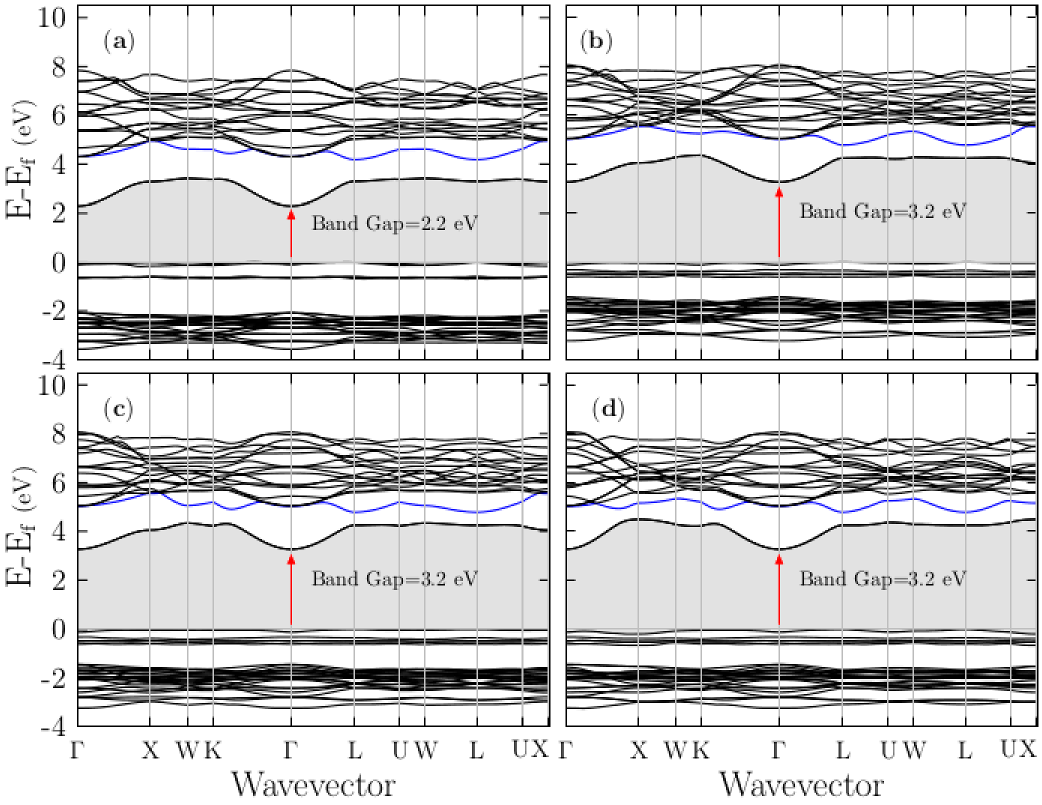

Figure S6. The upper panel (**a**,**b**,**c**,**d**) shows the calculated Kohn–Sham folded band structure of SC K_7Cl_8:Cu for the case of Figure S1(**a**,**b**,**c**,**d**) respectively and along with the same high-symmetry directions of the BZ used in the PC. The lowest direct Kohn–Sham bandgap, indicated by the red arrow, is 2.2 eV at the point for the case in Figure S1(**a**) and 3.2 eV. for the other three cases and indicated by the red arrow. The solid blue line depicts the second conduction band; notice there is a little change in its shape. The first conduction band shape almost keeps unchanged.

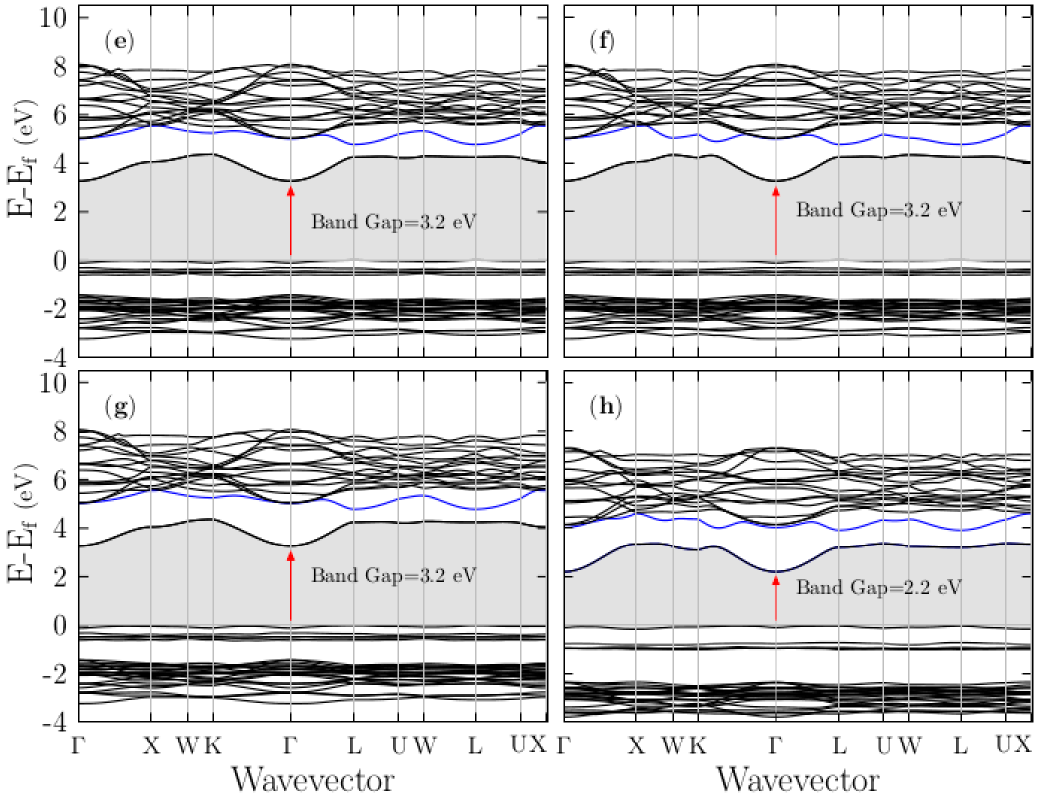

Figure S7. The upper panel (**e**,**f**,**g**,**h**) shows the calculated Kohn–Sham folded band structure of SC K_7Cl_8:Cu for the case of Figure S1 (**e**,**f**,**g**,**h**) respectively and along with the same high-symmetry directions of the BZ used in the PC. The lowest direct Kohn–Sham bandgap, indicated by the red arrow, is 3.2 eV at the point for the case in Figure S3 (**h**) and 3.2 eV for the other three cases and indicated by the red arrow. The solid blue line depicts the second conduction band; notice there is a little change in its shape. The first conduction band shape almost keeps unchanged. (only tiny changes).

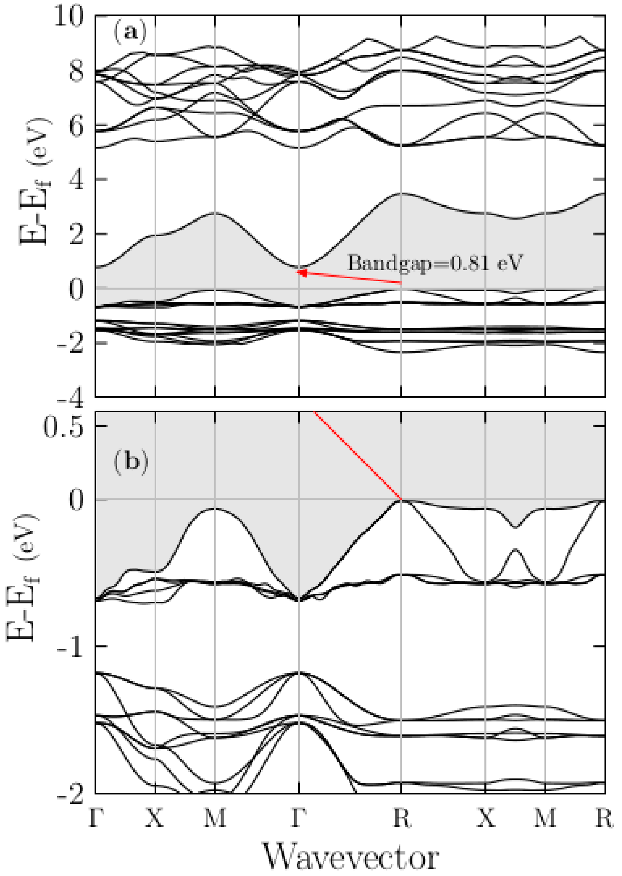

Figure S8. The upper panel (**a**) shows the folded bandstructure of the Cu doped supercell for KCl (CuK_3Cl_4) along the high-symmetry directions of the Brillouin zone (BZ) for a cubic cell. The lower panel (**b**) shows a zoomed view in the region −2 to 0.75 eV. The shortest indirect Kohn–Sham bandgap indicated by the red arrow is 0.80 eV between Γ and R points. Notice that the bandgap character changes from direct to indirect as the Cu density increases.
